# Changes in Diaphragmatic Function Induced by an Increased Inspiratory Load Experienced by Military Divers: An Ultrasound Study

**DOI:** 10.3389/fphys.2021.756533

**Published:** 2021-11-30

**Authors:** Sarah Rives, Bruno Schmid, Guillaume Chaumet, Fabienne Brégeon, Alain Boussuges

**Affiliations:** ^1^ERRSO, Institut de Recherche Biomédicale des Armées (IRBA), Toulon, France; ^2^Center for Cardiovascular and Nutrition Research (C2VN), INSERM, INRAE, Aix Marseille Université, Marseille, France; ^3^ALTRA BIO SA, Lyon, France; ^4^Service d’Explorations Fonctionnelles Respiratoires, CHU Nord, Assistance Publique des Hôpitaux de Marseille et Aix Marseille Univ, IRD, APHM, MEPHIIHU-Méditerranée Infection, Marseille, France

**Keywords:** respiratory muscle, diving, diaphragm, rebreather diving, chest ultrasonography

## Abstract

**Background:** Inspiratory loading is experienced by military divers when they use rebreather device. Our objective was to assess the changes in diaphragm function induced by an increase in inspiratory load at values similar to those experienced by divers in real life.

**Methods**: We recorded the excursion and the thickness of the right hemidiaphragm in 22 healthy male volunteers under inspiratory load conditions, using ultrasound in B- and M-mode. The measurements were performed at tidal volume and during breathing at 50% of inspiratory capacity. The breathing rate was regulated and similar in the various sessions with and without load.

**Results:** The rebreather device used by French military divers leads to an increase in inspiratory load of close to 30 cmH_2_O. Consequently, the session under load was performed using a device set to this threshold. Significant increases in the excursion and the thickening fraction of the diaphragm were observed between the sessions at tidal volume and at high volume. With addition of the inspiratory load, the excursion of the right hemidiaphragm increased significantly from 2.3 to 3.4cm at tidal volume and from 3.9 to 4.7cm at high volume. The thickening fraction increased significantly from 30.4 to 76.6% at tidal volume and from 70 to 123% at high volume. The statistical analysis demonstrated that assessment of the changes of the thickening fraction during breathing at tidal volume was the most relevant marker to assess the impact of the inspiratory load on the diaphragm.

**Conclusion:** Diaphragm ultrasound can be used to assess the changes in the diaphragm contraction pattern secondary to an increase in the respiratory load that can be generated by use a diving apparatus. The recording of the changes of the motion, and more importantly of the thickness of the diaphragm, during the breathing cycle is able to provide relevant information regarding the inspiratory load.

## Introduction

SCUBA diving leads to alterations in the work of breathing through the use of a gas mixture delivered by a regulator under hyperbaric conditions (Clarke and Flook, 1999). Ventilatory stressors are further increased during strenuous swimming. The changes in ventilatory load differ according to the position of the diver and the apparatus used. During a SCUBA diving ascent, when the diver is in an erect position, the hydrostatic pressure gradient between the regulator in the mouth and the lung centroid (i.e., the point of confluence of the forces exerted by the respiratory system) is negative (Lundgren, 1999; Krauza and Lundgren, 2007). This results in an increase in inspiratory load and a decrease in alveolar pressure (Castagna et al., 2018). Some divers are less able to tolerate an increased ventilatory workload, thus resulting in a risk of dyspnea and hyperventilation that exposes the diver to pulmonary barotrauma. Furthermore, through heart-lung interaction, the decrease in thoracic pressure induces an increase in the cardiac preload. The blood mass transfer toward the lungs and the heart can contribute to certain injuries such as water immersion pulmonary edema, particularly during strenuous exercise (Boussuges et al., 2017).

The increase in inspiratory load is all the more acute in military divers using a closed-circuit apparatus with a rebreathing bag on their back (the pressure gradient then becomes the pressure difference between the rebreather and the lung centroid) because the imbalance is present during the entire ventral kicking dive.

As the diaphragm supports the main part of the inspiratory workload, the diaphragmatic function can be modified during SCUBA diving. Assessing diaphragmatic function is difficult in healthy volunteers. The use of tools available for this purpose is limited due to either the risks associated with ionizing radiation and the need for transport (fluoroscopy) or the complex nature and the invasive characteristics of the test (measurement of transdiaphragmatic pressure; Minami et al., 2018). Over the past 25years, several studies have indicated that ultrasound is useful for morphological and functional assessment of the diaphragm (Ueki et al., 1985; Ayoub et al., 1997; Cohn et al., 1997; Boussuges et al., 2009). Various methods such as recording of the motion using M-mode ultrasonography or measurement of the changes of the diaphragmatic thickness during the breathing cycle have been proposed (for a review see Boussuges et al., 2020).

To assess the inspiratory stressors induced by a SCUBA breathing apparatus, analysis of the diaphragmatic function in healthy volunteers submitted to an increase in inspiratory resistance similar to that in a real SCUBA dive are likely to be informative. It has been reported, based on M-mode sonography, that a major increase in an inspiratory flow-resistive load can induce significant changes in the diaphragm contraction pattern, such as an increase in the inspiratory time and the diaphragm excursion (Soilemezi et al., 2013). However, in the work of Soilemezi et al. (2013), the volunteers were submitted to an inspiratory flow-resistive load equal to 50 cmH_2_O/l/s, which is higher than the stressors that may be experienced by divers in real life.

The first objective of this study was to assess the changes in diaphragmatic function induced by an increase in inspiratory load at values similar to those encountered by divers using a rebreather. The second aim of the study was to determine the most appropriate ultrasound parameters to analyze the impact of an increase in inspiratory load on the diaphragm.

## Materials and Methods

### Preliminary Study

**Figure 1:**
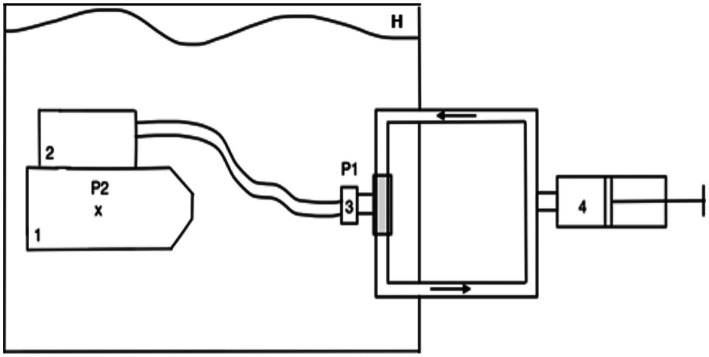
Principle of the measurements on the ventilator simulator. The mannequin and rebreather are immersed in water of a sufficient depth to preclude surface effects, but no deeper than 2m. 1=mannequin, x=lung centroid, 2=rebreather, 3=rebreather mouthpiece, 4=pump with valve for unidirectional flow in the circuit, P1 and P2=pressure sensors (differential pressure sensor), H=hyperbaric chamber.

To estimate the hydrostatic loading induced by the rebreathers used by the divers of the French Navy (Standard Complete Range Autonomous Breathing Equipment-STD CRABE, Aqualung, France), pressure-volume loops were recorded on a ventilator simulator (ANSTI LSTF 100m, JFD, United Kingdom). The operating principle of the simulator is depicted in Figure 1. The hydrostatic imbalance was measured using a differential pressure sensor, which provides a readout of the difference between the pressure measured at the lung centroid of the mannequin (location and normalized dimensions, measurement in water) and the pressure at the mouth of the mannequin (measurement in the ventilatory circuit).

### Main Study

In a previous study, Soilemezi et al. (2013) reported that when healthy volunteers were equipped with a mouthpiece and a nose clip, which leads to an increase in inspiratory resistance, the motion of the right hemidiaphragm increased from 1.7±0.5cm to 2.3±0.9cm (mean±SD) compared to no instrumentation. In our work, the calculation of the sample was based on this previous study. For an alpha risk of 0.05% and a power of 80%, we determined that at least 18 healthy volunteers would have to be included in the study.

The study was approved by the Regional Ethics Committee (Aix Marseille University, CPP-1, NoA01299-32) and all of the volunteers provided their written informed consent to participate in the experiment. The research was conducted according to the Helsinki Declaration regarding medical research involving humans.

Prior to the ultrasound measurements, a pulmonary function test was carried out in all of the healthy volunteers in order to determine their inspiratory capacity with spirometry maneuvers (Spirobank II Smart, MIR, Langlade, France).

### Protocol

Two sessions of the experiment were conducted: the volunteers were either submitted or were not submitted to a high inspiratory load, applied in a random order. The volunteers and the investigators were blinded to the inspiratory load used.

The volunteers were assessed in a seated position while breathing in a circuit equipped with a single-use mouthpiece, a disposable filter, and a commercially available threshold loading device (Philips Threshold IMT, Suresnes, Philips, France). For measurements without load, a tube of the same diameter and the same dead space (63ml) was used instead of the threshold loading device. Furthermore, a spirometer designed in our laboratory (Schmid and Boussuges, 2019) was placed at the end of the line. The spirometer was connected to a computer interface, which allowed the breathing rate and the gas volume to be measured continuously (breath-by-breath). The volunteers had real-time monitoring of inspired volume and breathing rate using a bar-graph. In order to ensure that the measured volumes were accurate, the spirometer was calibrated between each study phase, with the entire chain. Furthermore, this device was connected to the ultrasound machine *via* the electrocardiogram cables to record the signals of the beginning of both inspiration and expiration on the ultrasound images (Schmid and Boussuges, 2019).

The ultrasound measurements were performed during quiet breathing at tidal volume and during breathing at 50% of inspiratory capacity (deep breathing). The gas volumes were determined individually before the beginning of the study and a bar graph was used to guide the volunteers and to limit the variability of the breathing volume during the ultrasound examinations. The breathing rate and the volume were, therefore, similar between the two conditions involving load and no load.

### Ultrasound Examination

The study of diaphragmatic function was performed using an Esaote portable ultrasound system (Mylab 25CV, Genoa, Italy). Excursions and thickening of the right hemidiaphragm were studied.

#### Measurement of the Excursion of the Right Hemidiaphragm

The method used to record the diaphragmatic motion has been published (Boussuges et al., 2021). Briefly, the liver was used as an acoustic window to visualize the right hemidiaphragm. A 2–3.5MHz frequency probe (PA 230E Esaote, Genoa, Italy) was placed between the midclavicular and the middle axillary lines, below the right costal margin, and directed medially, cephalically, and dorsally so that the ultrasound beam was perpendicular to the posterior part of the vault. When the line of the M-mode could not be perpendicular to the cranial-caudal motion, M-anatomical mode was used. After correct viewing of the hemidiaphragm in two-dimensional mode (B-mode), M-mode was used to display the movement of the diaphragm along the selected line. The inspiratory and the expiratory cranio-caudal displacements of the diaphragm lead to a shortening and a lengthening, respectively, of the distance between the probe and the diaphragm. The diaphragmatic motion was assessed by M-mode while the patient breathed on tidal volume (quiet breathing) and at deep breathing.

#### Measurement of the Thickness of the Right Hemidiaphragm

The thickness of the diaphragm was measured at the area of apposition of the right hemidiaphragm to the rib cage under the costo-phrenic angle. A 7.5–12MHz frequency probe (LA 523 probe, Esaote, Genoa, Italy) was placed at the level of the 8th and 9th intercostal spaces on the anterior or the middle axillary line, which allows the diaphragm to be visualized between the pleural and peritoneal membranes. In accordance with current recommendations, measurement of the diaphragm thickness was performed from the middle of the pleural line to the middle of the peritoneal line (Carrillo-Esper et al., 2016) at the end of expiration and at the end of inspiration during rest ventilation, as well as during deep breathing.

### Measured Variables

The ultrasound parameters measured using M-mode included the excursion (cm), the duration (sec), and the velocity (cmsec^−1^) of the inspiratory motion. The parameters measured by B-mode included the thickness at end-inspiration, the thickness at end-expiration, and the thickening fraction (i.e., the ratio of the thickness at end-inspiration - the thickness at end-expiration divided by the thickness at end-expiration). All of the ultrasound parameters were recorded on the computer of the ultrasound machine for subsequent blind analysis. Measurements were obtained from the average of at least three different breathing cycles.

### Statistics

The data are expressed as mean±SD. The statistical tests were performed with R statistical software. The cohorts for comparison consisted of the healthy volunteers during two sessions, with and without inspiratory load, at two respiratory regimens, i.e., tidal volume and 50% inspiratory capacity.

Comparison between the continuous variables was carried out with the analysis of variance. If the distribution of the residuals was not normal, a log10 transformation was performed on response variable Y and then a two-way ANOVA was computed with the transformed response variable. *Post hoc* analyzes were performed when needed with Holm correction. The interaction effect (breathing volume: load) was studied to determine the ultrasound parameters related to the changes in breathing conditions. Differences between groups were considered significant at *p*<0.05.

## Results

### Preliminary Study

**Figure 2:**
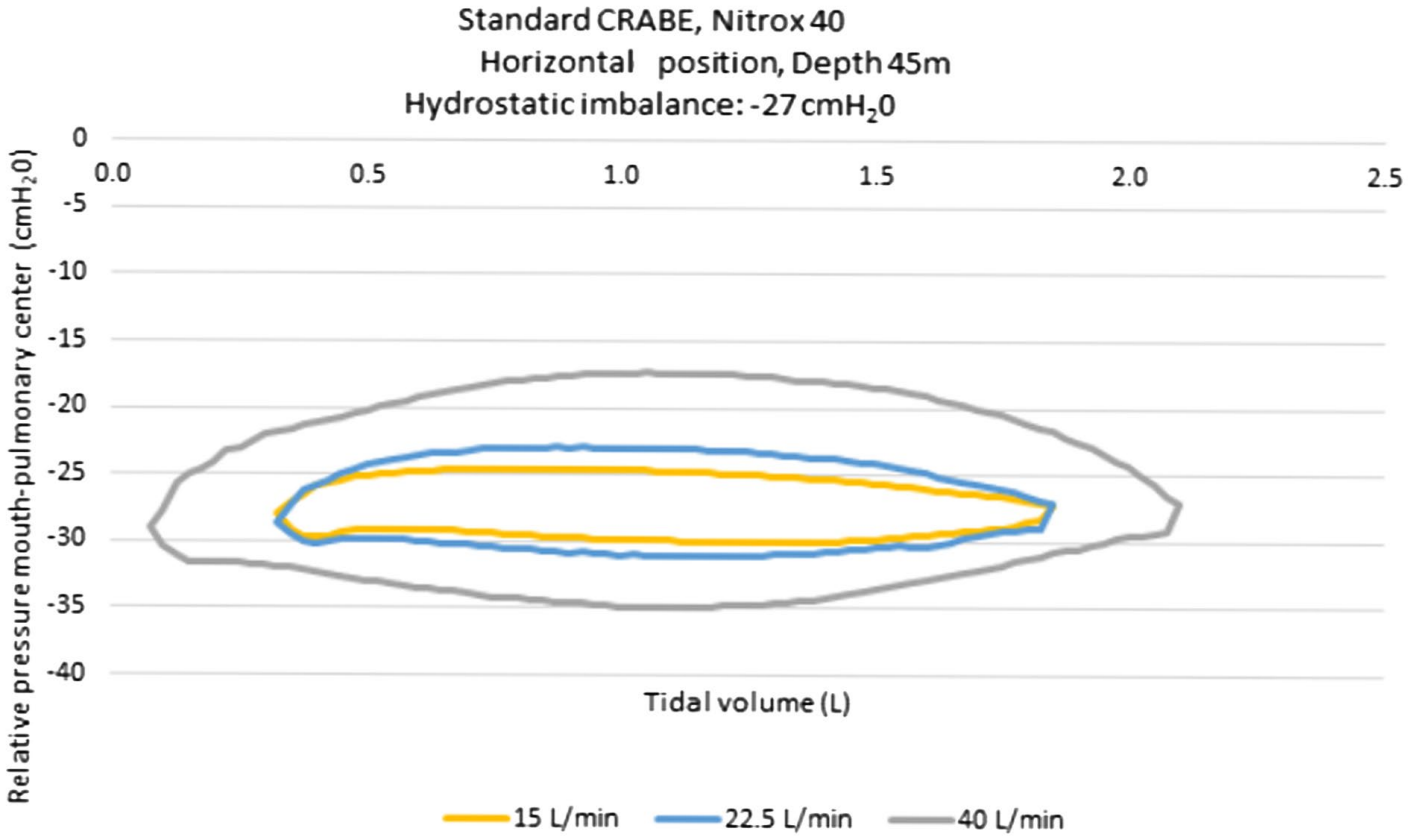
Pressure-volume loops obtained with the standard CRABE rebreather on a ventilatory simulator, at a 45-meter depth under three ventilatory flow conditions.

The pressure-volume loops recorded on the ventilator simulator are presented in Figure 2. The loops are in principle centered on the pressure 0 when measuring the work of breathing. We shifted them by the value of the hydrostatic imbalance measured on the rebreather in a horizontal position (the devices are carried on the diver’s back). This value was measured at −27mbar (cmH_2_O). The maximal inspiratory pressure varied according to the ventilation output: −30 cmH_2_O for 15l/min, −31 cmH_2_O for 22.5l/min and−35 cmH_2_O for 40l/min. According to the ventilator regimen of the volunteers (quiet breathing and increase in the breathing volume at 50% of inspiratory capacity) the inspiratory load, chosen for the experiment, was −30 cmH_2_O.

### Main Study

In total, 22 healthy male adult volunteers were included in this study. The mean age, weight and height were 34±9years, 73±6kg, 175±5cm, respectively.

Images of the right hemidiaphragm were successfully obtained from the 22 healthy volunteers. Their average inspiratory capacity was determined to be 3.7±0.6l.

During the entire experiment, the breathing rate was similar and individually regulated depending on the respiratory parameters recorded during quiet breathing. It comprised 12 to 16 breaths per minute. The mean tidal volume was 0.7±0.1l leading to a ventilation output of 8.5±1.2l/min. During the session at 50% of inspiratory capacity, the breathing volume and the ventilation output were 1.9±0.3l and 24±6l/min, respectively.

During the session at tidal volume, the inspiratory resistance induced by the threshold set at 30 cmH_2_O was calculated to be 31±10 cmH_2_O/l/s using the formula delta P/dV/dt according to the Poiseuille’s law with delta *p* =30 cmH_2_O, DV=tidal volume and dt=inspiratory time.

This calculation was a simple estimate. Indeed, the increase in inspiratory load is known to alter the flow, especially at high volume, leading to a turbulent flow. To calculate airway resistances using Poiseuille’s law, the flow should be laminar. Therefore, we have chosen to report the estimate of inspiratory resistance at tidal volume, only.

#### Statistical Study

**Figure 3:**
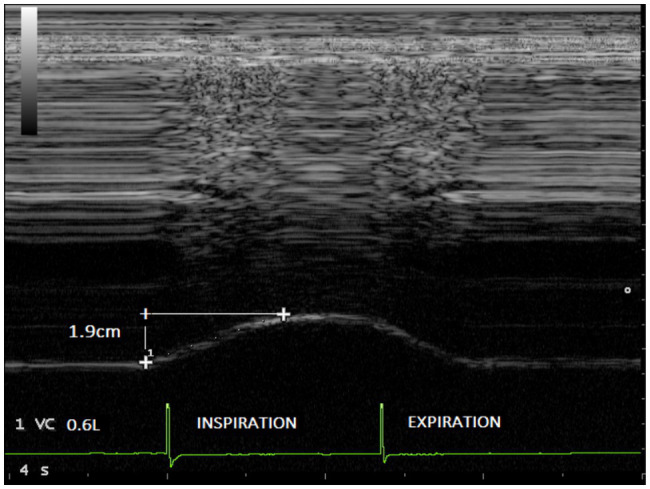
Diaphragmatic excursion recorded in one volunteer during breathing at tidal volume (0.6l) without load (1.9cm).

**Figure 4:**
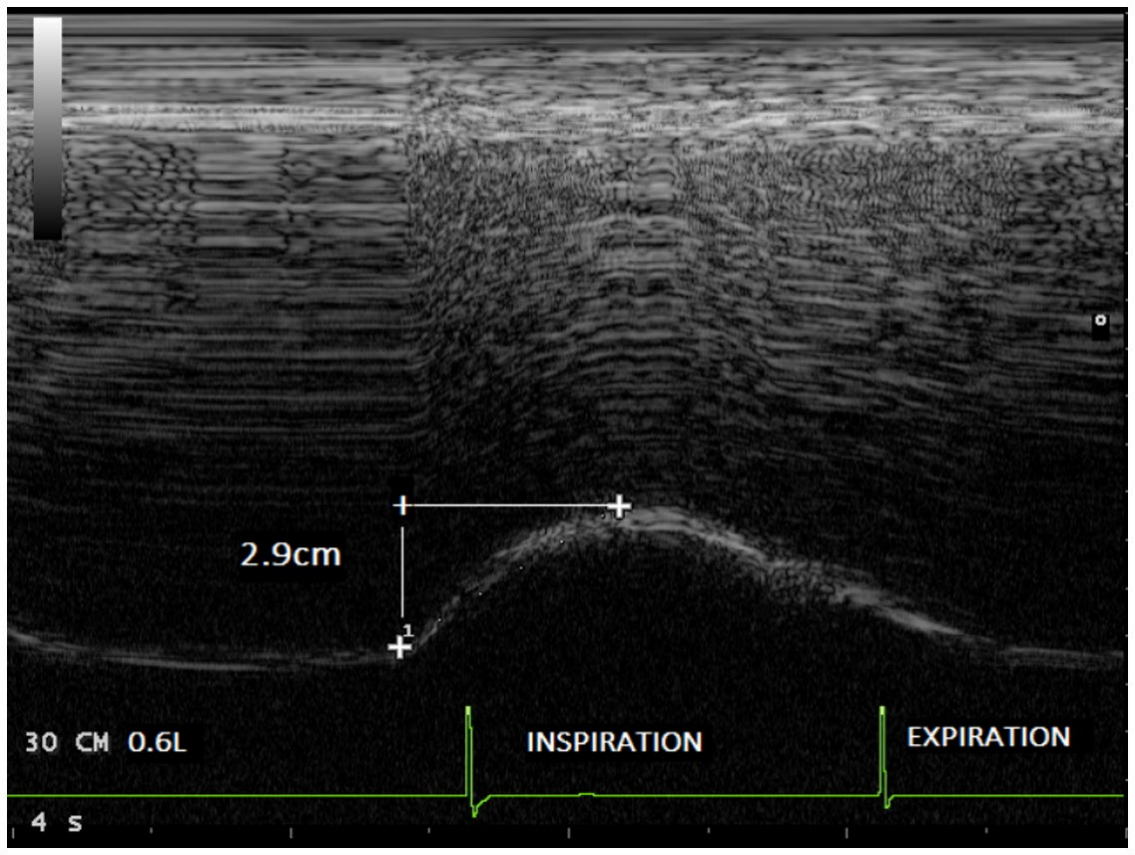
Diaphragmatic excursion recorded in the same volunteer during breathing at tidal volume (0.6l) with an inspiratory load of 30 cmH_2_O (2.9cm).

**Figure 5:**
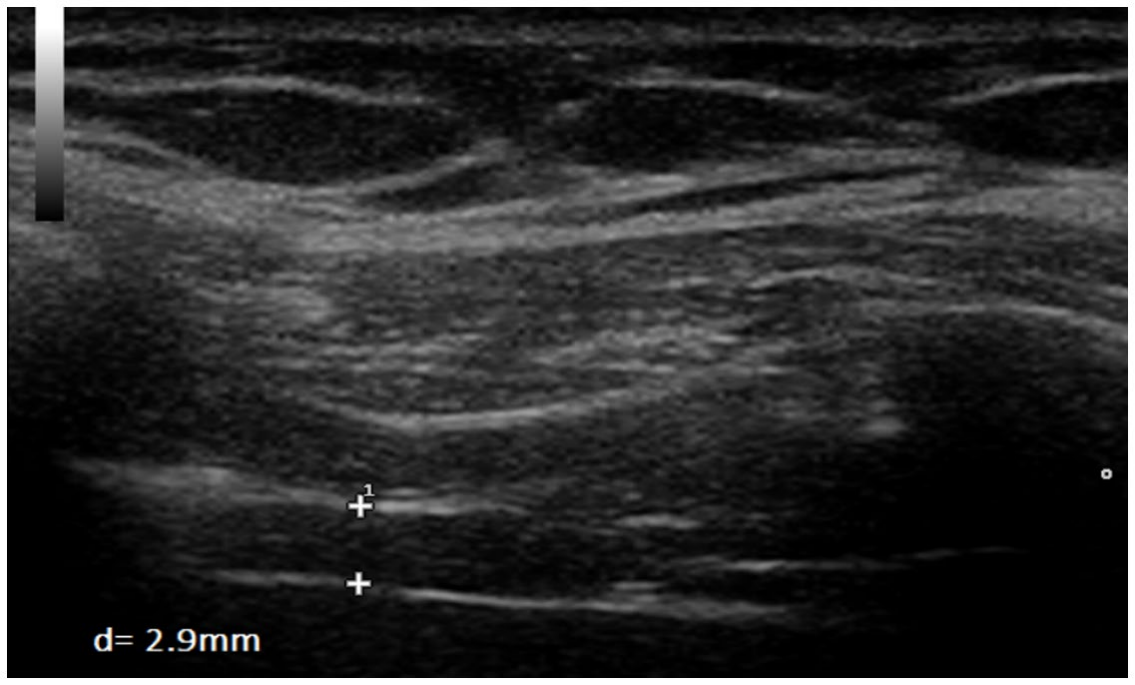
Measurement of diaphragm thickness in one volunteer at end-inspiration without load (d=2.9mm).

**Figure 6:**
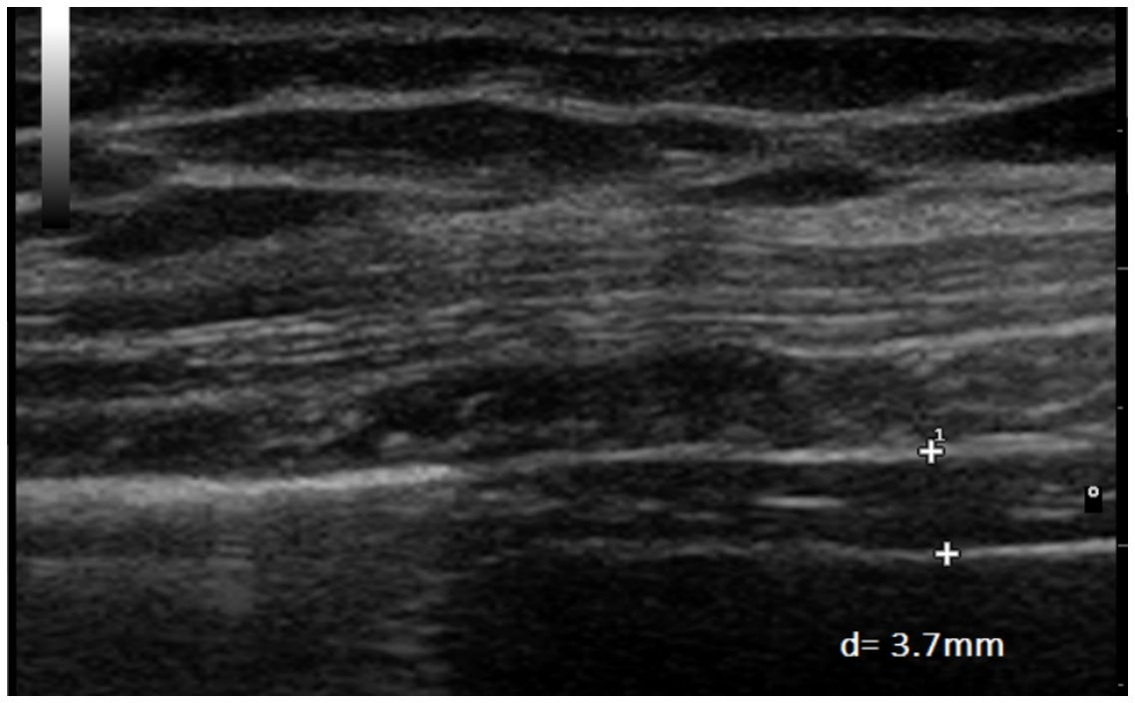
Measurement of diaphragm thickness in the same volunteer at end-inspiration with an inspiratory load of 30 cmH_2_O (*d*=3.7mm).

The end expiratory thickness did not vary significantly, at any of the examination time points. Irrespective of the session (without load and with a load of 30 cmH_2_O), significant increases in both the excursion (Figures 3, 4) and the thickening fraction (Figures 5, 6) were recorded in the measurements performed when the volunteers breathed at 50% of inspiratory capacity compared to tidal volume.

**Figure 7:**
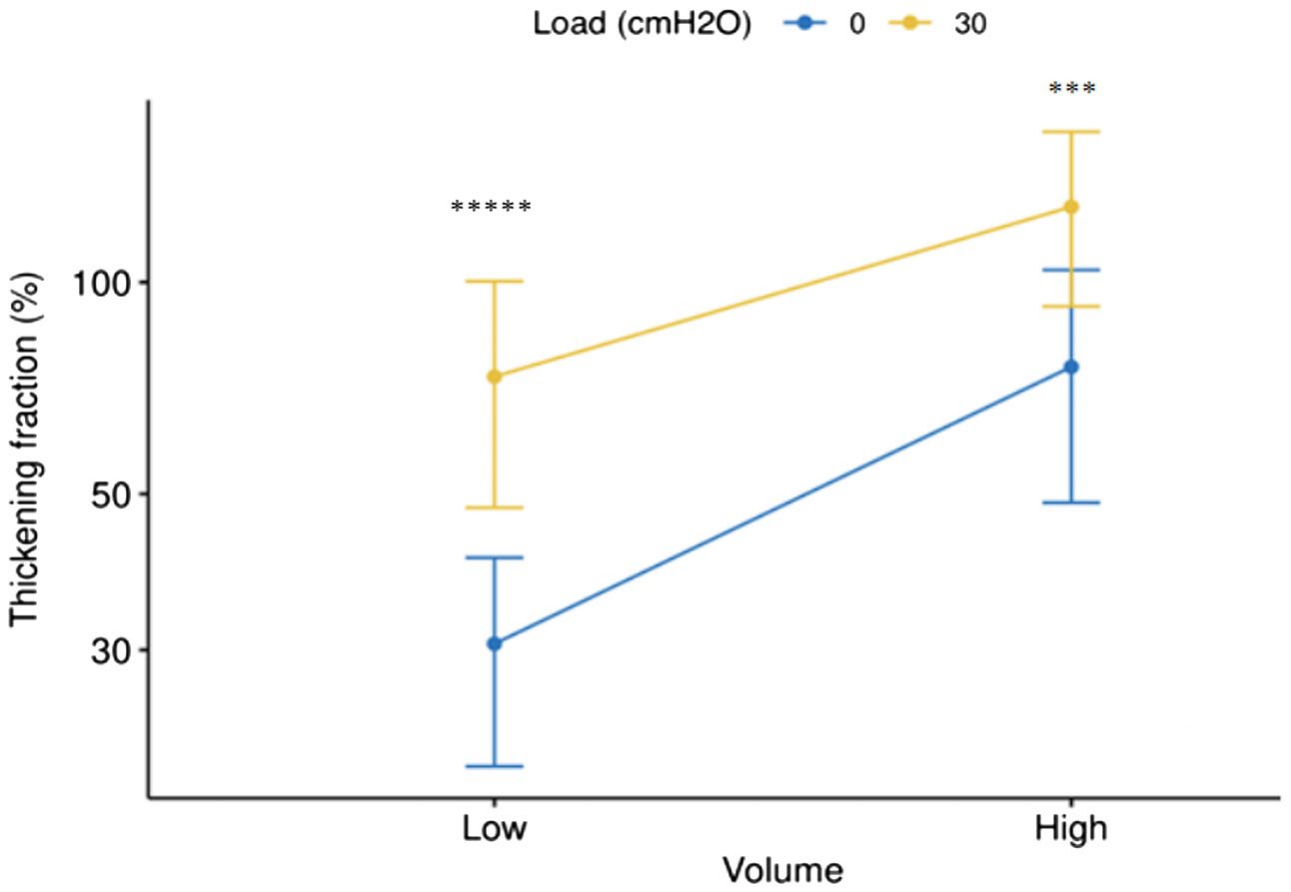
Error bar (Mean±SD) of changes of thickening fraction of right hemidiaphragm (Log10) in the sessions with and without load at low (tidal volume) and high volume (50% of inspiratory capacity). ^*****^p <10^−11^, ^***^p <0.001.

Tables 1, 2 and Figure 7 report the changes induced by the addition of the inspiratory load in volunteers breathing at tidal volume and at 50% inspiratory capacity, respectively.

**Table 1:** Changes in the diaphragmatic parameters induced by a high inspiratory load in volunteers breathing at tidal volume.

	Without load	Load 30 cmH_2_O	p
	Mean±SD	
Excursion (cm)	2.3±0.5	3.3±0.9	<0.001
Inspiratory time (sec)	1.5±0.5	1.6±0.7	NS
Inspiratory velocity (cmsec^−1^)	1.6±0.5	2.3±0.8	<0.002
Thickness at end-expiration (mm)	2.1±0.5	2.1±0.4	NS
Thickness at end-inspiration (mm)	2.7±0.6	3.6±0.8	<0.001
Thickening fraction (%)	31±12	73±23	<0.001

**Table 2:** Changes in the diaphragmatic parameters induced by a high inspiratory load in volunteers breathing at high volume.

	Without load	load 30 cmH_2_O	p
	Mean±SD	
Excursion (cm)	4.1±1	4.8±0.9	0.01
Inspiratory time (sec)	1.8±0.5	2±1.1	NS
Inspiratory velocity (cmsec^−1^)	2.6±1.3	2.9±1.4	NS
Thickness at end-expiration (mm)	2.1±0.5	2±0.4	NS
Thickness at end-inspiration (mm)	3.7±1.1	4.6±1	<0.005
Thickening fraction (%)	76±26	128±39	<0.001

The interaction effect (breathing volume: load) was significant on the thickening fraction (%) [*F*(1,84)=0.11, *p* value=0.029] only (Tables 3, 4). This means that the thickening fraction was the better parameter to assess the impact of the changes in breathing volume and inspiratory load.

**Table 3:** Study of the interaction effect (breathing volume: load).

	F	value of p
Excursion (cm)	*F* (1.84)=1.03	NS
Inspiratory time (sec)	*F*(1.84)=0.14	NS
Inspiratory velocity (cm.sec-1)	F (1.84)=0.07	NS
Thickness at end-expiration (mm)	F (1.84)=0	NS
Thickness at end-inspiration (mm)	F (1.84)=0	NS
Thickening fraction (%)	F (1.84)=0.11	<0.05

**Table 4:** Type III ANOVA results for the thickness fraction.

Parameter	Sumsq	df	Statistic	Value of p
(Intercept)	75.464	1	3.326	<0.001
Breathing volume (low-high)	1.691	1	74.557	<0.001
Inspiratory load (without-30 cmH_2_O)	0.622	1	27.425	<0.001
Volume:load	0.112	1	4.936	<0.05
Residuals	1.905	84		

## Discussion

The main finding of this study is that an increase in the inspiratory load that can be experienced by military divers leads to an increase in the excursion and the thickening fraction of the right hemidiaphragm. Our statistical analysis retained assessment of the thickening fraction during quiet breathing as the best parameter for accurate assessment of the consequences of an increase in inspiratory resistance on diaphragmatic function.

Previous studies have shown that various parameters such as the physical effect of water immersion (Moon et al., 2009), the increase in the breathing gas density (Held and Pendergast, 2013), and the pressure difference between the regulator and the lung centroid (the negative static lung load; Lundgren, 1999) lead to an increase in the work of breathing during SCUBA diving. The inspiratory static lung loading has been estimated to be approximately −25 cmH_2_O in a diver in a prone position under water using a closed-circuit apparatus with a rebreathing bag on their back (at a lower hydrostatic pressure than the lung centroid; Lundgren, 1999). The increase in inspiratory load led to a more negative pleural pressure that was variably estimated as a function of the diver, the ventilatory regimen, the depth, and the diving apparatus. In our study, the level of the inspiratory load was based on the recording of the pressure-volume loops of the rebreather used by French military divers on a respiratory simulator. The inspiratory load chosen for the experiment was −30 cmH_2_O according to the ventilation output of the volunteers. We have studied two ventilatory regimens, i.e., breathing at tidal volume and at 50% of inspiratory capacity, to simulate an increase in ventilation output induced by swimming. As expected, we observed increased excursions at high volume compared to tidal volume. This has been reported previously in studies performed in healthy volunteers (Cohen et al., 1994; Houston et al., 1994). For example, Houston et al. (1994) recorded cranio-caudal excursions of the posterior part of each hemidiaphragm on successive respiratory cycles in 14 healthy subjects. Spirometric measurements were recorded simultaneously on a spirometer. The authors observed a linear relationship between the diaphragmatic excursion and the inspired volumes.

During breathing against an elevated inspiratory load, an increase in the right hemidiaphragm excursion was found compared to breathing without resistance. This difference was significant at tidal volume and at high volume. Furthermore, at tidal volume, the velocity of the diaphragm was increased. These findings were suggested by previous works. Ayoub et al. (1997) found an increase in the duration and the amplitude of the diaphragmatic motion during an increase in inspiratory resistance induced by the addition of a spirometric chain in 8 subjects. Furthermore, Soilemezi et al. (2013) reported an increase in diaphragm excursion with the addition of low resistance (mouthpiece) or high resistance (inspiratory resistance at 50cm H_2_O/l/s) compared to breathing without a device. In contrast to our findings, at high resistance, they found a decrease in the breathing rate that led to an increase in the inspiratory time and a decrease in the inspiratory velocity.

The discrepancies between studies can be explained by a number of differences between the protocols. On the one hand, the inspiratory resistance was lower in our work (estimated mean of 31±10cm H_2_O/l/s). On the other hand, we had set a fixed breathing rate for all phases of the experiment. The regulation of the breathing rate was chosen to make it easier to record the differences between the ultrasound markers of diaphragm contractility in the two conditions. However, this led to a degree of deviation from the conditions encountered in real life. This could have affected the overall respiratory adaptation, in particular the changes in the inspiratory time and the velocity.

Our statistical analysis revealed that the thickening fraction was increased at high volume compared to tidal volume. This increase was the result of an increase in the diaphragm thickness at the end of inspiration. Furthermore, as expected and according to previous studies that reported a good reproducibility of this measurement (Cohn et al., 1997; Matamis et al., 2013; Goligher et al., 2015), the same thickness was recorded at the end of expiration. The increase in the thickening fraction at high volume was not surprising since a relationship was previously found between the thickening fraction and the inspiratory volume measured by spirometry (Goligher et al., 2015).

The thickening fraction was increased under inspiratory load both at tidal and at high volume.

During breathing at a high inspiratory load, the increase in diaphragmatic work has been demonstrated using a number of different procedures. Transdiaphragmatic pressure requires simultaneous recordings of esophageal pressure and gastric pressure, and it is calculated as the differential pressure between these two signals (Minami et al., 2018). Abdominal pressure usually increases during inspiration whereas esophageal pressure decreases as this reflect the lowering of the pleural pressure. It has been reported that transdiaphragmatic pressure increased progressively in COPD patients from a low to a high intensity of threshold load training (from 30 to 80% of maximal inspiratory pressure), based on use of a Philips Threshold IMT (Wu et al., 2017). In such circumstances, the increase in diaphragm activity has been also demonstrated by the neural respiratory drive measured from the diaphragm electromyogram (Jolley et al., 2008; Wu et al., 2017). Previous studies have reported that some ultrasound parameters correlated with physiological measurements of diaphragm strength. Ueki et al. (1985) reported a strong correlation between the maximum inspiratory pressure and the thickening fraction. Cardenas et al. (2018) found that the diaphragm mobility and the thickening fraction were related to the inspiratory muscle strength assessed by maximum inspiratory pressure or transdiaphragmatic pressure. Goligher et al. (2015) have shown that the thickening fraction correlated with the electrical activity recorded by diaphragm electromyography and transdiaphragmatic pressure, although the correlation coefficient was low. With a high inspiratory load, it is thought that the extra diaphragmatic inspiratory muscles including the external intercostal, sternocleidomastoid and scalene muscles are solicited in addition to the increase in the activity of the diaphragm (de Troyer et al., 2005; Hudson et al., 2007). This can explain the discrepancies between studies and the fact that some authors have reported a lack of or a low correlation between the ultrasound marker of diaphragm activity and inspiratory load (Goligher et al., 2015; Oppersma et al., 2017).

To assess the changes in diaphragm function induced by an increase in inspiratory load that can be experienced by military divers, our statistical analysis demonstrated that the more sensitive parameter should be the measurement of the thickening fraction. This result is in keeping with the study of Umbrello et al. (2015) performed in intensive care unit. These authors reported that diaphragm thickening was a better indicator of respiratory effort than diaphragm excursion.

The purported relevance of the use of a high ventilation output to detect the changes in the thickening fraction in volunteers submitted to inspiratory loading is not supported by our results. Indeed, although the breathing rate was the same, a change in the respiratory pattern including an increase in inspiratory velocity was observed at tidal volume, during the session with load in comparison with the session without load (see Table 1). This difference was not observed when volunteers breathed at high volume (see Table 2). Furthermore, the difference in the thickening fraction between the two sessions was more significant at tidal volume than at high volume (see Figure 7). Consequently, the study of high volumes does not appear to bring any additional interest. Therefore, to assess the respiratory stressors induced by the use of a diving apparatus, it can be recommended to study the changes in the diaphragm thickening fraction during quiet breathing, which is easier to perform and better tolerated by the volunteers.

### Study Limits and Perspectives

Our study used a model that reproduced an inspiratory load close to the resistance experienced by SCUBA divers, on the basis of measurements made by a ventilator simulator on a rebreather used by French Navy. It would be relevant to repeat the study in divers using their rebreather with the common stressors experienced during open-water diving, i.e., water immersion and a hyperbaric environment. Thanks to the progress in technologies, such a study is a realistic prospect. Indeed, it was recently demonstrated that it is possible to assess the cardiac function of a diver using underwater Doppler-echocardiography at a 10-meter depth in a swimming pool (Marabotti et al., 2013). In further works, to assess the contribution of the changes in the respiratory function on the risk of pulmonary edema, it would be useful to record hemodynamic data by ultrasound in combination with assessment of the respiratory function. Using these methods, it would be possible to compare the cardio-respiratory impact of the various apparatuses used by divers.

In contrast to previous studies, in our work, the inspiratory resistances were not calculated individually according to the maximal inspiratory pressure. Consequently, during inspiratory load, the work of breathing, as a percentage of the maximal capacity, could differ between individuals according to their respiratory capacity. This method was chosen because the level of inspiratory resistance was determined based on the measurement of a ventilator simulator. This inspiratory load is experienced by military divers irrespective of their level of fitness and their respiratory capacity.

In the present study, the thickening fraction appeared to be a better marker of an increase in diaphragm activity under inspiratory load compared to diaphragm excursion. In a recent study, Oppersma et al. (2017) suggested that the assessment of the diaphragm using speckle-tracking imaging should be more informative than conventional ultrasound. They reported that speckle-tracking parameters such as the strain and the strain rate correlated better with transdiaphragmatic pressure and electric activity of the diaphragm than the thickening fraction. Consequently, in further studies, it would be interesting to use speckle-tracking imaging to assess the changes in diaphragm function induced by dive apparatuses such as rebreathers.

## Conclusion

An inspiratory load similar to the level experienced by military divers using a rebreather led to an increase in both the excursion and the thickening fraction of the right hemidiaphragm.

Diaphragm ultrasound is a promising tool for assessment of the impact of dive apparatuses on diaphragmatic function. In this context, the more relevant parameter appeared to be assessment of the changes in the thickening fraction during breathing at tidal volume.

## Data Availability Statement

The raw data supporting the conclusions of this article will be made available by the authors, without undue reservation.

## Ethics Statement

The studies involving human participants were reviewed and approved by Aix Marseille University, CPP-1, NoA01299-32. The patients/participants provided their written informed consent to participate in this study.

## Author Contributions

AB and FB conceived and designed the study. SR and BS assisted with the technical aspects of the protocol, recruited all the participants, and were involved in the acquisition of the data. AB and SR performed the ultrasound examinations. AB and GC analyzed the data and performed the statistical analysis. AB, GC, and SR have drafted the article while FB and BS revised it critically for important intellectual content. All authors have given final approval of the version to be published.

## Funding

This study was supported by a French Ministry of Defense research grant (Direction Générale de l’Armement, PDH-1-SMO-2-1).

## Conflict of Interest

The authors declare that the research was conducted in the absence of any commercial or financial relationships that could be construed as a potential conflict of interest.

## Publisher’s Note

All claims expressed in this article are solely those of the authors and do not necessarily represent those of their affiliated organizations, or those of the publisher, the editors and the reviewers. Any product that may be evaluated in this article, or claim that may be made by its manufacturer, is not guaranteed or endorsed by the publisher.
